# The feasibility of using pedometers and brief advice to increase activity in sedentary older women – a pilot study

**DOI:** 10.1186/1472-6963-8-169

**Published:** 2008-08-08

**Authors:** Jacqui A Sugden, Falko F Sniehotta, Peter T Donnan, Paul Boyle, Derek W Johnston, Marion ET McMurdo

**Affiliations:** 1 Section of Ageing & Health, Division of Medicine & Therapeutics, University of Dundee, Ninewells Hospital & Medical School, Dundee, UK; 2 School of Psychology, University of Aberdeen, King's College, Old Aberdeen, Aberdeen, UK; 3 Tayside Centre for General Practice, Community Health Sciences, MacKenzie Building, University of Dundee, Dundee, UK; 4 School of Geography and Geosciences, University of St Andrews, Irvine Building, North Street, St Andrews, UK

## Abstract

**Background:**

People over the age of 70 carry the greatest burden of chronic disease, disability and health care use. Participation in physical activity is crucial for health, and walking accounts for much of the physical activity undertaken by sedentary individuals. Pedometers are a useful motivational tool to encourage increased walking and they are cheap and easy to use. The aim of this pilot study was to evaluate the feasibility of the use of pedometers plus a theory-based intervention to assist sedentary older women to accumulate increasing amounts of physical activity, mainly through walking.

**Methods:**

Female participants over the age of 70 were recruited from primary care and randomised to receive either pedometer plus a theory-based intervention or a theory-based intervention alone. The theory-based intervention consisted of motivational techniques, goal-setting, barrier identification and self-monitoring with pedometers and daily diaries. The pedometer group were further randomised to one of three target groups: a 10%, 15% or 20% monthly increase in step count to assess the achievability and acceptability of a range of targets. The primary outcome was change in daily activity levels measured by accelerometry. Secondary outcome measures were lower limb function, health related quality of life, anxiety and depression.

**Results:**

54 participants were recruited into the study, with an average age of 76. There were 9 drop outs, 45 completing the study. All participants in the pedometer group found the pedometers easy to use and there was good compliance with diary keeping (96% in the pedometer group and 83% in the theory-based intervention alone group). There was a strong correlation (0.78) between accelerometry and pedometer step counts i.e. indicating that walking was the main physical activity amongst participants. There was a greater increase in activity (accelerometry) amongst those in the 20% target pedometer group compared to the other groups, although not reaching statistical significance (p = 0.192).

**Conclusion:**

We have demonstrated that it is feasible to use pedometers and provide theory-based advice to community dwelling sedentary older women to increase physical activity levels and a larger study is planned to investigate this further.

## Background

People aged 70 years and over, are the fastest growing segment of the Scottish population and carry the greatest proportion of chronic disease burden, disability and health care use [1]. It is known that participation in regular physical activity is crucial for health and function in later life [1], yet achieving greater participation in physical activity remains a major public health challenge.

Walking accounts for a substantial portion of the energy expenditure associated with physical activity in sedentary individuals. We targeted sedentary individuals for behaviour change interventions as this is the group with the most to gain in terms of health and functioning from an increase in physical activity [2,3], mainly through walking. Walking is often reported as their preferred leisure time activity [4].

Behaviour change interventions should be based on theory [5]. Self-regulation theory emphasises the role of goal setting, planning and self-monitoring in behaviour change [6]. Goal planning and self-monitoring of behaviour are crucial for behaviour change. Self monitoring allows a comparison between current walking behaviour and the goal behaviour thus indicating when adjustments are necessary. Self-monitoring of walking behaviour could be challenging as walking is often less structured and salient than planned physical activities, however self-monitoring aids such as pedometers can allow for timely and accurate self-monitoring of walking behaviour. Pedometers are easy to use, provide feedback which has important informational and motivational effects and have been shown to be accurate in, and acceptable to community dwelling older adults [7,8]. In this pilot study the use of pedometers was combined with an intervention based on self-regulation theory. A recent systematic review of 48 studies found that interventions that promoted walking could increase physical activity of sedentary individuals and that people could be motivated to do more walking when interventions were tailored to their needs [9]. Pedometers have been shown to be effective at enhancing interventions aimed at increasing physical activity levels [10-12].

The primary aim of this pilot study was to establish whether it was possible to use pedometers together with a theory-based intervention, individualised activity plans and diary keeping in assisting sedentary older women to accumulate increasing amounts of physical activity, mainly through walking. We also sought to determine the type of pedometer (spring levered or piezoelectric) that was more suited to our study population and to assess the achievability of the target increases in step count. To determine whether walking accounted for the main proportion of physical activity of this population, we aimed to correlate accelerometer counts (physical activity) and pedometer counts (walking) at baseline for those participants in the pedometer group. In addition we also sought to determine adherence with diary keeping. Secondary outcome measures were lower limb function, health related quality of life, anxiety and depression.

## Methods

The Tayside Committee on Medical Ethics approved the study which was carried out in accordance with the Declaration of Helsinki. Written, informed consent was obtained.

Participants were seen in their own homes on three occasions. Study visits lasted approximately an hour. At the first visit, consent was taken, randomisation took place and the participant was asked to wear an accelerometer on the hip during waking hours for 7 days (a validated device [13] for measuring physical activity, RT3 Tri-axial Research Tracker, Stay Healthy Inc., USA). The self regulation intervention was carried out at the second visit (7 days later) along with collection the secondary outcome data. Outcome measures at the end of the study were collected on the third and final visit, three months later.

### Inclusion and exclusion criteria

Women aged over 70 years who were insufficiently active or sedentary, i.e. no participation in moderate-intensity physical activity of at least 30 minutes at least 5 days per week or at least 20 minutes of continuous vigorous-intensity activity three or more times a week [14] were included in the study. Women were asked about their participation in physical exercise and walking at a preliminary phone call before any visits took place. Women fulfilling physical activity recommendations, resident of institutional care, housebound (unable to increase outdoor walking), having moderate to severe cognitive impairment (MMSE score < 18) precluding informed consent, having significant visual impairment and so unable to read pedometer count screen, wheelchair bound or unwilling to participate were excluded from the study.

### Recruitment and randomisation

**Figure 1:**
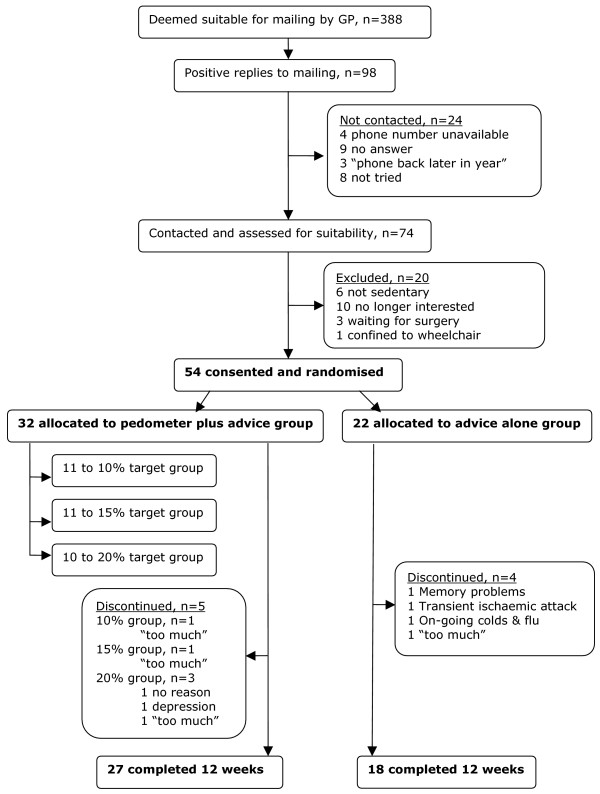
Consort diagram.

Participants were recruited from a single GP practice (see figure 1) via the well established Scottish Primary Care Research Network (SPCRN). The GP principal provided a list of all women aged 70 years and over, excluding those who should not be approached because of terminal illness, recent bereavement, severe heart failure/COPD/dementia or nursing home dwellers. The GP wrote to the women inviting them to take part in the study, including a pre-paid reply envelope. Those accepting the invitation were telephoned and asked whether they were housebound, how much exercise they were taking, whether they were visually impaired or using a wheelchair. At the first face-to-face visit, a mini mental state examination was performed.

Randomisation was performed by an individual not otherwise involved in the study using a computer-based random number generator. The participants were allocated at random, in a 3:2 ratio, to one of two groups: pedometer plus self-regulation intervention ("pedometer group") or self-regulation intervention alone ("advice group"). The pedometer group were further randomised to one of three target groups: a 10%, 15% or 20% monthly increase in step count (steps taken per day). The average daily pedometer count over three consecutive days (at baseline before intervention) was taken and used to set a target of achieving a 10%, 15% or 20% increase in steps during the first month. If the participant met her target step count, it was increased again after the first and second months. If she had not met her target, 10%, 15% or 20% was added to the average number of steps they achieved over the three day period preceding the telephone call.

### Development of the self-regulation intervention and protocol for delivery

Self-regulation theory emphasises the role of goal setting, planning and self-monitoring in behaviour change [14]. Theory-based advice was given to each participant in the form of individualised activity action plans and coping plans. Additional file 1 shows the protocol that was developed for the delivery of this advice. The protocol was developed using the *Coding Manual to Identify Behaviour Change Techniques in Published Intervention Descriptions *[15]. The intervention was delivered by a senior research nurse who had received training from two experienced health psychologists (authors DJ and FFS). First, each participant was given advice about the health benefits of increasing physical activity both verbally and in pamphlet form after collection of baseline data at the second visit. Then action plans and coping plans were discussed and written with each participant in her own home after the baseline data had been collected. The action plans were designed to increase participants' physical activity levels (mainly through walking) and the coping plans were to identify how to cope with possible barriers towards increasing their walking [16]. A graded approach to increasing walking was given with clear advice on when and where to walk and how to schedule time for physical activity. Each participant was given a daily activity diary to complete with logs of either pedometer count or time spent walking outdoors. These diaries were returned to the researcher, in a supplied stamped addressed envelope, to assess compliance with diary keeping. Each participant was contacted by telephone once a week for the first month and then fortnightly thereafter until the end of the study to provide motivation, encouragement and to troubleshoot any problems.

### Validation of pedometers

Two different types of pedometer were used in this study in order to ascertain how accurate and user-friendly they were in our study population: the Omron HJ-005 spring levered and the Omron HJ-113 piezoelectric pedometers. The spring levered device is worn on the waistband; a horizontal lever-arm moves up and down in response to vertical movement and opens and closes an electrical circuit. This device must remain vertical to be effective [17]. In this study the piezoelectric device was worn around the neck although it can be worn on the waistband. It contains a horizontal cantilevered beam with a weight at the end that compresses a piezoelectric crystal when subjected to movement, it is less susceptible to tilt than the spring levered device [17]. At the randomisation visit (visit 2), each participant in the pedometer group was asked to read the count screen of each device and to walk 100 steps at their usual pace wearing both pedometers in the presence of the researcher. They were also asked which device they preferred. The more accurate device was given to the participant and they were asked to wear it to monitor their step count during waking hours.

### Outcome measures

The outcome measures were assessed at baseline (before the intervention) and after 3 months. Outcome measures were collected on the same day on both occasions and baseline outcomes were assessed before implementation of the intervention. The primary outcome measure was change in daily activity levels which was measured by accelerometry (RT3 Tri-axial Research Tracker, Stay Healthy Inc., USA), a device the size of a pager worn on the waistband during waking hours for a 7-day period which has been validated for use in an elderly population [13]. Accelerometers were worn for 7 days by the participant, tri-axial data was collected in 1 minute epochs, counts < 250 or > 3000 were discarded as spurious [18]. Counts were totalled over each 24 hr period (midnight – midnight), the 1st set of 24 hour data was discarded (incomplete day) and missing days were excluded from analysis. Counts (per minute) per day for valid days were recorded.

Secondary outcome measures were: assessment of lower limb function using a validated performance score [19] consisting of three 0–4 point scales summarising performance on three tests of lower extremity function – usual walking speed over 3 meters, standing balance and repeated chair stands; health related quality of life was assessed using the EuroQuol questionnaire which provides a brief measure of health status with self ratings on mobility, self-care, anxiety and depression, usual activities and pain together with a global rating of health state which has been shown to be reliable at interview in an older population [20]; and depression and anxiety were assessed using the Hospital Anxiety and Depression Scale [21]. This pilot study was not powered to detect changes in outcome measures, but to demonstrate feasibility of data collection for a larger trial.

### Statistical analysis

Descriptive information was generated for readability of the pedometer screen, accuracy of step counting for the two pedometer types, adherence with daily activity diaries and drop out rate. The correlation between pedometer counts and accelerometry at baseline was examined using Pearson's correlation (2 tailed), and the difference in change in accelerometer count and secondary outcomes from visit 1 to visit 3 between the two groups was analysed by t-test assuming that variances were not the same. Comparisons between target groups were carried out using analysis of variance followed by Bonferroni corrected pair-wise tests. In addition, a test for trend across target groups was carried out. Where variables were non-Normally distributed, non-parametric tests such as Mann-Whitney were utilised. All analyses were implemented in SPSS.

## Results

54 participants (mean age 76, range 70–86), confirmed as sedentary or insufficiently active, were recruited into the study from a single GP practice (see figure 1).

### Feasibility of using pedometers and brief advice to increase activity in sedentary older women

There was 100% adherence with activity action plan and coping plan completion (nobody refused). Diary filling and measurement of all the outcome measures was acceptable to all participants. Compliance with diary completion was 96% in the pedometer plus advice group, with one diary that went missing and an average of 2 day's entries per participant missing over the whole 3 month study period. In the advice alone group, compliance with dairy completion was lower at 83% with 4 diaries that went missing and an average of 5 day's entries per participant missing.

9 participants dropped out of the study in total (17%). 5 participants dropped out from the pedometer group: 1 from the 10% target group (felt the study was "too much"), 1 from the 15% target group (study "too much"), and 3 from the 20% target group (1 gave no reason, 1 had depression, 1 felt study was "too much"). 4 participants dropped out from the advice group (1 felt study was "too much", 1 had memory problems, 1 had a transient ischaemic attack and 1 had on-going colds and flu), see figure 1.

### Validation of pedometers for this study population

All participants in the pedometer group were able to read the count screens on both pedometers. The piezoelectric pedometer was generally preferred over the spring levered device by the participants because it recorded step counts more accurately in the target population; the spring levered pedometer counted an average of 62 steps per 100 steps taken (range 1–179), the piezoelectric pedometer counted an average of 73 steps per 100 steps taken (range 6–117). There were complaints of over-counting from several participants using spring levered device and we received no complaints from the participants using the piezoelectric device. The piezoelectric device has a 7-day inbuilt memory which allowed the researcher to confirm pedometer counts noted by the participants. The majority of participants preferred the piezoelectric device. 12 participants used the spring levered device and 20 participants used the piezoelectric. Daily step counts for all participants in the pedometer group were low at baseline, with an average of 2895 steps per day (range 185 to 8845).

### Accelerometry and pedometer counts

There was a strong correlation of 0.78 (p = 0.01, Pearson's correlation) between accelerometry and pedometer step counts at baseline, i.e. time spent walking, for both pedometer types combined.

**Figure 2:**
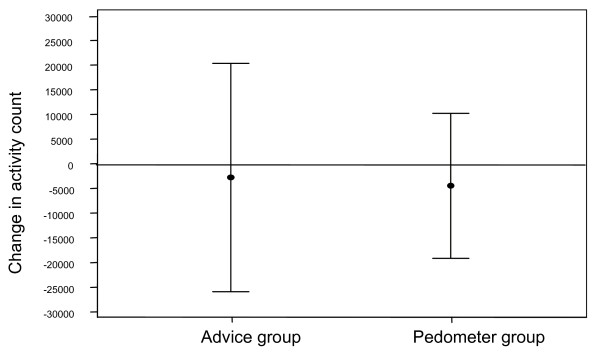
Change in activity between baseline and 12 weeks in theory-based intervention alone group compared to pedometer group as a whole, 95% confidence intervals shown.

There was no difference in change in accelerometry count between the advice group and the pedometer group as a whole (t-test, p = 0.893, table 1 and figure 2), however, there was an increase in activity (accelerometry) amongst those in the 20% target pedometer group compared to the other target groups although not reaching statistical significance (p = 0.192). Looking at the daily diary entries, the advice group met the target 58% of days compared with 61% of days in the pedometer group (p < 0.001). In the pedometer group, the 10% target group met the target 55%, 15% target group 57% and 20% target group 74%. There was a higher rate of meeting the target in the highest target group.

**Table 1:** Change in accelerometry (daily activity count) results in pedometer group as a whole and intervention alone group, n = 44^†^. Accelerometry (daily activity count) results, n = 44^†^

Time	Intervention Group	N	Mean	Standard Deviation	Standard Error Mean	p-value*
Baseline	Advice group	18	116,378	47,952	11,302	0.816
	Pedometer group	26	112,984	46,887	9,195	
12 weeks	Advice group	18	113,822	62,337	14,693	0.776
	Pedometer group	26	108,738	54,728	10,733	
Change (12 weeks – baseline)	Advice group	18	-2,556	46,494	10,958	0.893
	Pedometer group	26	-4,245	36,355	7,129	

† 1 missing accelerometry data

* t-test assuming variances not same

### Secondary outcomes

There was no difference in change in quality of life (EuroQuol questionnaire) between the two groups. There was no significant difference in change in depression (Hospital Anxiety and Depression Scale, HADS) between the two groups although there was a greater reduction in the pedometer group. Change in anxiety over the duration of the study was higher in the pedometer group (p = 0.021). Limb function improved in the pedometer group relative to the advice group (table 2). Most of the outcomes were approximately normally distributed, although costs and EQ5D were significantly non-normal. Therefore medians and interquartile ranges are also shown for each variable.

**Table 2:** Change in secondary outcomes (positive indicates increase in value), n = 45

Group		EQ5D score	EuroQuol scale	HADS Anxiety	HADS Depression	Health costs	Limb function
Advice N = 18	Mean	0.047	1.83	-1.056	-1.056	-80.93	0.361
	Standard Deviation	0.151	18.24	2.577	1.731	681.44	1.370
	Median	0.000	0.00	-0.500	-1.000	-7.60	0.000
	IQR	0.091	23.0	2.250	2.250	172.70	2.250

Pedometer N = 22	Mean	0.014	2.81	0.445	-1.000	-51.47	0.611
	Standard Deviation	0.132	19.39	1.649	1.981	180.59	1.303
	Median	0.000	5.00	0.000	-2.000	-3.60	1.000
	IQR	0.142	20.00	2.000	2.000	127.00	1.000

Mann-Whitney	p-value	0.645	0.824	0.021*	0.769	0.577	0.385

* Significant, p < 0.5

Higher score = greater anxiety, greater depression, better quality of life, increased health costs and better limb function.

## Discussion

Sedentary older women were selected as our target population because they have the most to gain from increasing their activity levels [2,3]. Interventions to promote walking have been shown to increase physical activity in sedentary individuals [9] and the Women's Health and Ageing Study showed that even small amounts of regular walking can confer protection from further mobility loss [22]. In this subgroup of the population, walking is often the preferred leisure time activity [4] and structured group activity sessions may hold limited appeal for older adults [23]. Theory based interventions have been effective at increasing physical of older individuals [24,25].

In this pilot study we examined the feasibility of using pedometers plus a theory-based intervention and diary keeping to increase physical activity levels mainly through walking in sedentary older women living in the community. We have demonstrated that it is practical to recruit sedentary older women through primary care via the well established Scottish Primary Care Research Network (SPCRN). By writing to all eligible subjects in a single GP practice we were able to quantify the proportion of eligible subjects on the practice list, the proportion who were sedentary and the proportion of sedentary older women approached consenting to participate. Studies of physical activity will only attract the willing, the advantage of our study over much of th rest of the literature in the area is that we have not recruited by advertisement which produces an even more biased sample. Media campaigns have been criticised for resulting in the recruitment of highly motivated, non-representative individuals [26]. By recruiting via primary care, our study is able to report on the total eligible population as well as those actually accepting the invitation to take part.

Two different types of pedometer were used to assess which was most suited to the target population. We found the piezoelectric device to be the more accurate device for this study population and this is supported by current literature advocating accuracy of piezoelectric pedometers for individuals with slower walking speeds including older people [27]. Whilst spring levered pedometers have been found to be accurate above speeds of 3 miles per hour, piezoelectric pedometers are more sensitive at slower speeds [27] and have also been found to be more accurate in those who are obese or overweight [17]. Pedometers have been shown to be beneficial tools for increasing physical activity in a number of studies [28,10-12].

We found that there was good correlation between pedometer step count and accelerometry. This is in keeping with previous work which demonstrated both that a simple pedometer can provide a good estimate of physical activity [29] and that walking accounts for a substantial proportion of physical activity in sedentary individuals [4]. We have demonstrated an encouraging trend towards an increase in walking amongst participants using pedometers with a 20% monthly increase target, however, as a pilot study, it was not powered to detect significant changes in activity between groups. This will be investigated in a subsequent larger trial. The drop out rate of this study (17%) compares favourably with a physical activity behavioural change study, also recruiting from primary care but with a lower age (40.6 years), which reported a drop out rate of 21% [30]. There is a dearth of previous similar work in older people with which to compare our pilot findings.

We have demonstrated good adherence with diary filling and measurement of all the outcome measures was acceptable to all participants, and whilst the study was not powered to detect changes in outcomes but we have shown it is feasible to collect the data. We were surprised to find a significant increase in anxiety in the pedometer group as the feedback that we had from the participants indicated that most of them enjoyed taking part and felt, at the end of the study, that they were doing more walking. The subsequent planned large randomised trial will investigate this observation further.

This pilot study had limitations which will be addressed in the main study. The researcher collected data and implemented the intervention giving the potential for observer bias. We noticed a marked measurement affect with accelerometry; more effort was made when first wearing the accelerometer. We will address this in the larger trial by asking the participants to wear the accelerometer for 14 days and discarding the first 7 days of data. Seasonality may have affected the results of the pilot. Work by the authors is ongoing to investigate this and it will be addressed in the main study. Modest numbers of participants were involved in the pilot, whereas in the main study we will recruit 210 participants.

## Conclusion

In conclusion, the feasibility of using pedometers and brief advice as a practical approach to increasing activity (mainly through walking) in sedentary older women has been demonstrated in this pilot study and merits further investigation. Most physical activity interventions to date have focussed on younger adults, but the over 65s constitute the fastest growing, but most sedentary, segment of the UK population and effective strategies to achieve increased participation in physical activity remain a major public health challenge. This pilot study has allowed us to develop and test the feasibility of using the self-regulation intervention, and we are now conducting a large randomised trial to examine the effectiveness of pedometers to increase physical activity levels/walking in sedentary older women.

## Competing interests

The authors declare that they have no competing interests.

## Authors' contributions

METM, DWJ, PB, PTD and FFS conceived and designed the study. JAS collected the data. PTD analysed the data. JAS and METM wrote the paper. All authors edited, revised and approved the final manuscript.

## Pre-publication history

The pre-publication history for this paper can be accessed here:



## Supplementary Material

Additional file 1 Box 1. Self-Regulation Intervention – Protocol for delivery of systematic advice. Click here for file
