# Supplementary material for: The feasibility of using pedometers and brief advice to increase activity in sedentary older women – a pilot study
Source: BMC Health Serv Res. 2008 Aug 8;8:169. doi: 10.1186/1472-6963-8-169 (PMC2527003; doi:10.1186/1472-6963-8-169)
Supplement: Additional file 1 — Box 1. Self-Regulation Intervention – Protocol for delivery of systematic advice. [file 1472-6963-8-169-S1.doc]

(1) Information will be provided to the subject about the link between increasing the amount of walking they do and better health;

Walking is good for you. You can enjoy walking any time, alone or with a friend, and it’s free.

(2) A leaflet will be given to the subject containing useful tips for walking.

(3) Information will be provided about the consequences (benefits and costs) of doing more walking;

By increasing the amount you walk, you will:

● feel good● lower your blood pressure

● protect yourself from mobility loss● increase your fitness

● control your body weight● boost your energy levels

● make your bones stronger

By increasing the amount you walk, there is a risk of:

● becoming tired, especially if you do too much too quickly.

(4) A discussion will be initiated about others’ approval;

How would your family feel if you did more walking?

How would your doctor feel if you did more walking?

(5) Subjects will be prompted to think about the support they receive from significant others;

Tell your friends and family about the study, they will help and encourage you.

(6) The subjects’ intention to do more walking will be established and subjects will be encouraged to think about how they might incorporate more walking into their weekly schedules;

Now that you have decided to increase the amount you walk, we will make a plan together about how to change your routine so that you can do more walking.

(7) Subjects will be prompted set themselves specific goals: detailed plans of what they will do. It is important to emphasize that these plans are a useful tool for increasing walking [17,18].

(8) Subjects will be prompted to think about potential barriers to these plans and how they might overcome them. Internal barriers, such as lack of motivation, will be emphasized in a way that does not make them feel abnormal.

(9) Monthly targets will be set, in steps or minutes depending on which group they are randomised to. These targets will increase by 15% each month for the first three months of the study;

You will increase the amount you walk gradually by setting monthly targets.

(10) Subjects will be asked to keep a daily diary and record all walking activity, in steps or minutes, depending on which group they are randomised to;

Keeping a daily diary of your walking will help you to stick to your plans.

(11) Subjects will receive regular telephone calls (once a week for the first month and fortnightly thereafter) during which the researcher will provide feedback on their performance and general encouragement.
